# Association between systemic immune-inflammation index, systemic inflammation response index, and adverse outcomes in aneurysmal subarachnoid hemorrhage: a meta-analysis

**DOI:** 10.3389/fneur.2025.1596126

**Published:** 2025-10-06

**Authors:** Yihong Hao, Jianfang Zhao, Jie Han, Dajiang Hao

**Affiliations:** Department of Neurosurgery, The Third People’s Hospital of Datong, Datong, Shanxi, China

**Keywords:** aneurysmal subarachnoid hemorrhage, systemic inflammation response index, systemic immune index, meta-analysis, prognosis

## Abstract

**Purpose:**

This systematic review seeks to explore the link of the systemic immune-inflammation index (SII) and systemic inflammation response index (SIRI), to adverse outcomes (AOs) in aneurysmal subarachnoid hemorrhage (aSAH). The findings may provide scientific evidence to enable the timely identification of patients at high risk and guide the formulation of personalized treatment strategies.

**Patients and methods:**

PubMed, Cochrane, Embase, and Web of Science databases were comprehensively retrieved from inception to August 10, 2024. The Newcastle-Ottawa Scale (NOS) was leveraged for appraise the quality of the encompassed studies. Statistical analyses were undertaken via Stata 17.0, and a bivariate mixed-effects model was applied for diagnostic value evaluation.

**Results:**

Sixteen studies involving 4,510 aSAH individuals were encompassed. The meta-analysis demonstrated SII as a predictor of poor 90-day outcomes [Odds Ratio (OR) = 1.94, 95% Confidence Interval (CI): 1.35–2.78, *p* < 0.001], with an area under the receiver operating characteristic (ROC) curve (AUC) of 0.77 (95% CI: 0.73–0.81), sensitivity of 0.71 (95% CI: 0.61–0.79), and specificity of 0.72 (95% CI: 0.62–0.80). Additionally, SII can be utilized for forecasting delayed cerebral ischemia (DCI) (OR = 1.39, 95% CI: 1.35–1.43, *p* < 0.001), with an AUC of 0.80 (95% CI: 0.76–0.83), sensitivity of 0.75 (95% CI: 0.56–0.88), and specificity of 0.73 (95% CI: 0.62–0.82). SIRI was also found to predict unfavorable 90-day outcomes (OR = 1.19, 95% CI: 1.09–1.30, *p* < 0.001), with an AUC of 0.79 (95% CI: 0.75–0.82), sensitivity of 0.66 (95% CI: 0.58–0.73), and specificity of 0.76 (95% CI, 0.73–0.80). However, SIRI was not a significant predictor of DCI (OR = 1.37, 95% CI: 0.94–2.02, *p* = 0.105) or postoperative pneumonia (POP) (OR = 3.73, 95% CI: 0.68–20.35, *p* = 0.128).

**Conclusion:**

Both SII and SIRI serve as predictive biomarkers for unfavorable 90-day outcomes in the aSAH population, with SII also demonstrating predictive value for DCI. While both indices exhibit moderate accuracy, further research is necessitated to validate their clinical utility.

**Clinical trial registration:**

https://www.crd.york.ac.uk/PROSPERO/, Registration No: CRD42024585116.

## 1 Introduction

Aneurysmal subarachnoid hemorrhage (aSAH) is a prevalent condition in neurosurgery and its annual incidence rate is nearly 1.3%. The cumulative incidence rates over 10, 20, and 30 years are reported to be 10.5, 23.0, and 30.3%, respectively (1). Currently, endovascular embolization is generally considered the first-line treatment due to its minimally invasive nature and higher safety profile. Craniotomy with aneurysmal clipping is reserved for cases where endovascular intervention is contraindicated or deemed unsuitable (2). However, postoperative outcomes in aSAH patients vary considerably; while patients with mild presentations may achieve complete recovery, those with severe conditions face high rates of mortality and disability (3, 4). Timely intervention, prevention of complications, and systematic rehabilitation are critical for improving prognosis. Identifying prognostic factors influencing aSAH outcomes has consistently been a focus of clinical research. Reliable predictive markers can assist clinicians in implementing effective, individualized therapeutic strategies for different patient populations.

In recent years, mounting evidence has proved the critical influence of systemic inflammatory responses on determining the prognosis and survival of the aSAH population (5–7). Following the onset of aSAH, blood components such as red blood cell breakdown products and hemoglobin come into contact with cerebrospinal fluid, activating both local and systemic immune cells, thereby triggering a cascade of inflammatory responses (8). These inflammatory processes are not confined to the central nervous system but extend systemically, as reflected by significant changes in markers like the systemic immune-inflammation index (SII) and systemic inflammation response indexv (SIRI) (9, 10).

The SII is an integrated marker for assessing immune status and inflammation levels, calculated via the formula: (neutrophil count × platelet count)/lymphocyte count. It is a promising tool for gaging the host’s inflammation and immune equilibrium (11). Risen SII levels are linked to adverse prognoses in various cardiovascular diseases (12, 13). Similarly, in aSAH patients, higher SII values have been linked to more severe neurological deficits, increased incidence of delayed cerebral ischemia (DCI), and less favorable clinical outcomes (14). The systemic inflammation response index (SIRI) is a newly identified biomarker based on circulating immune cells, calculated as (neutrophil count × monocyte count)/lymphocytes (15). It systematically reflects the intricate interplay and possible synergistic influence among neutrophils, monocytes, and lymphocytes in the microenvironment, thereby showing the equilibrium between inflammatory and immune responses in the body. SIRI has been proven to be a useful marker for evaluating immune function (16). In aSAH patients, SIRS frequently occurs and has been associated with more severe brain injury, prolonged hospitalization, and higher mortality rates (17).

Although the prognostic value of SII and SIRI in the aSAH cohort was clarified, systematic reviews have not been undertaken for evidence consolidation and a holistic summary of findings. Therefore, our study comprehensively evaluates the efficacy of SII and SIRS as prognostic indicators in aSAH via systematic review and meta-analysis. Our ultimate goal is to present scientific evidence to enable the early detection of high-risk people and the formulation of customized curing strategies in clinical practice.

## 2 Materials and methods

Our study rigorously followed the Preferred Reporting Items for Systematic Reviews and Meta-Analyses (PRISMA) 2020 (18) and was registered on the PROSPERO platform following the literature search (Registration No.: CRD42024585116).

### 2.1 Search strategy

Two independent researchers comprehensively retrieved the PubMed, Cochrane, Embase, as well as Web of Science, covering pertinent studies from inception to August 10, 2024 on the predictive significance of SII and SIRI in people suffering from aSAH. The search involved Medical Subject Headings (MeSH) and free-text terms. Keywords included “Subarachnoid Hemorrhage,” “SIII,” “SIRI,” and their relevant variations. Our search strategies are presented in Supplementary Table S1. Additionally, the reference lists of pertinent articles were manually checked to guarantee that no relevant studies were overlooked.

### 2.2 Eligibility criteria

Our eligibility criteria were defined as per the PICOS framework (19).

The inclusion criteria were:

(1) The subjects of this study were adult patients (≥18 years) with aneurysmal subarachnoid hemorrhage, confirmed by computed tomography (CT) or lumbar puncture, and documented by angiography or computed tomographic angiography (CTA).
(2) Exposure Factors: High levels of SIRI or SII.
(3) Control Group: Low levels of SIRI or SII.
(4) Outcomes: Studies providing sufficient data to directly or indirectly estimate the association between SIRI or SII and adverse outcomes (AOs) at 90 days, DCI risk [odds ratios (ORs) with 95% confidence intervals (CIs)], and diagnostic accuracy (sensitivity, specificity, or raw data for constructing 2 × 2 contingency tables, including true positives, false positives, true negatives, and false negatives). AOs were examined via the modified Rankin Scale (mRS) and the Glasgow Outcome Scale (GOS). A good prognosis was denoted as mRS < 3 or GOS scores of 4–5, whereas a poor prognosis was indicated as mRS ≥ 3 or GOS scores of 1–3 (20–22).
(5) Study Design: Observational studies—case–control and retrospective cohort studies.

The exclusion criteria were:

(1) Reviews, case reports, meta-analyses, or conference abstracts; (2) *In vitro* or animal studies; (3) Replicate publications; (4) Those without full texts or sufficient information for ORs and 95% CIs calculation; (5) Non-English publications; (6) Studies utilizing the same patient cohort as other included studies.

The research utilizing the most extensive dataset was chosen when multiple studies relied on identical data sources to avoid duplication.

### 2.3 Literature screening and data extraction

As per the predefined eligibility criteria, two researchers independently screened the retrieved literature. The retrieved records were imported into EndNote 21 to remove duplicates. The titles and abstracts of the deduplicated records were then reviewed for preliminary screening. Articles that met the preliminary criteria were subsequently retrieved in full and assessed to determine final eligibility. The consistency of the screening results between the two researchers was evaluated using the Kappa statistic; a Kappa value greater than 0.8 indicated good agreement. The two researchers then independently extracted data, including the first author’s name, publication year, age, country, type of indicator, source institution of the study population, and male sample size, and performed cross-checking of the extracted data. In cases where discrepancies arose regarding study inclusion, exclusion, or data extraction, and consensus could not be reached through discussion, a third researcher was consulted to arbitrate, thereby ensuring consistency of decisions and accuracy of the data.

### 2.4 Quality assessment

Two researchers gaged the study quality independently via the Newcastle-Ottawa Scale (NOS) (23) and conducted subsequent cross-checking. In case of disagreements, a third researcher provided adjudication. The NOS evaluates three domains across eight items: four for study participant selection, one for group comparability, and three for outcome evaluation. Each item, except for comparability (which can score up to two points), scores 1 point at maximum, yielding a total score of 0–9. Higher scores suggest better study quality, with 7–9 denoting high quality. Study selection, data extraction, and quality assessment were conducted independently by two researchers. Both investigators have experience in systematic reviews, extensive training in clinical research methodology, and prior participation in meta-analyses. Before commencing this study, all team members received training in Cochrane systematic review methodology.

### 2.5 Data analysis

Data analysis was undertaken through Stata 17.0 (StataCorp LLC, College Station, TX). The statistical models were chosen based on the heterogeneity index (I^2^). A random-effects model was employed if I^2^ surpassed 50%. Otherwise, a fixed-effects model was utilized. When heterogeneity was substantial, sensitivity and subgroup analyses were carried out to identify the possible sources. Publication bias across and within studies was visually assessed via funnel plots. Egger’s test was leveraged for detecting statistical publication bias whose influence on our meta-analysis results was examined via the trim-and-fill approach.

A bivariate mixed-effects model was utilized to unveil the prognostic value. Point estimates and corresponding 95% CIs for sensitivity, specificity, positive likelihood ratio (PLR), negative likelihood ratio (NLR), and diagnostic odds ratio (DOR) were computed for every group. The summary receiver operating characteristic (SROC) curve was derived. The area under the curve (AUC) and its 95% CI, were determined. The presence of threshold effects was checked via Spearman’s correlation coefficient, and corresponding p (24). *p* > 0.05 indicated no threshold effect-related heterogeneity across studies. Deeks’ funnel plot asymmetry test helped with publication bias detection and *p* < 0.05 signified statistical significance.

## 3 Results

### 3.1 Literature search

**Figure 1:**
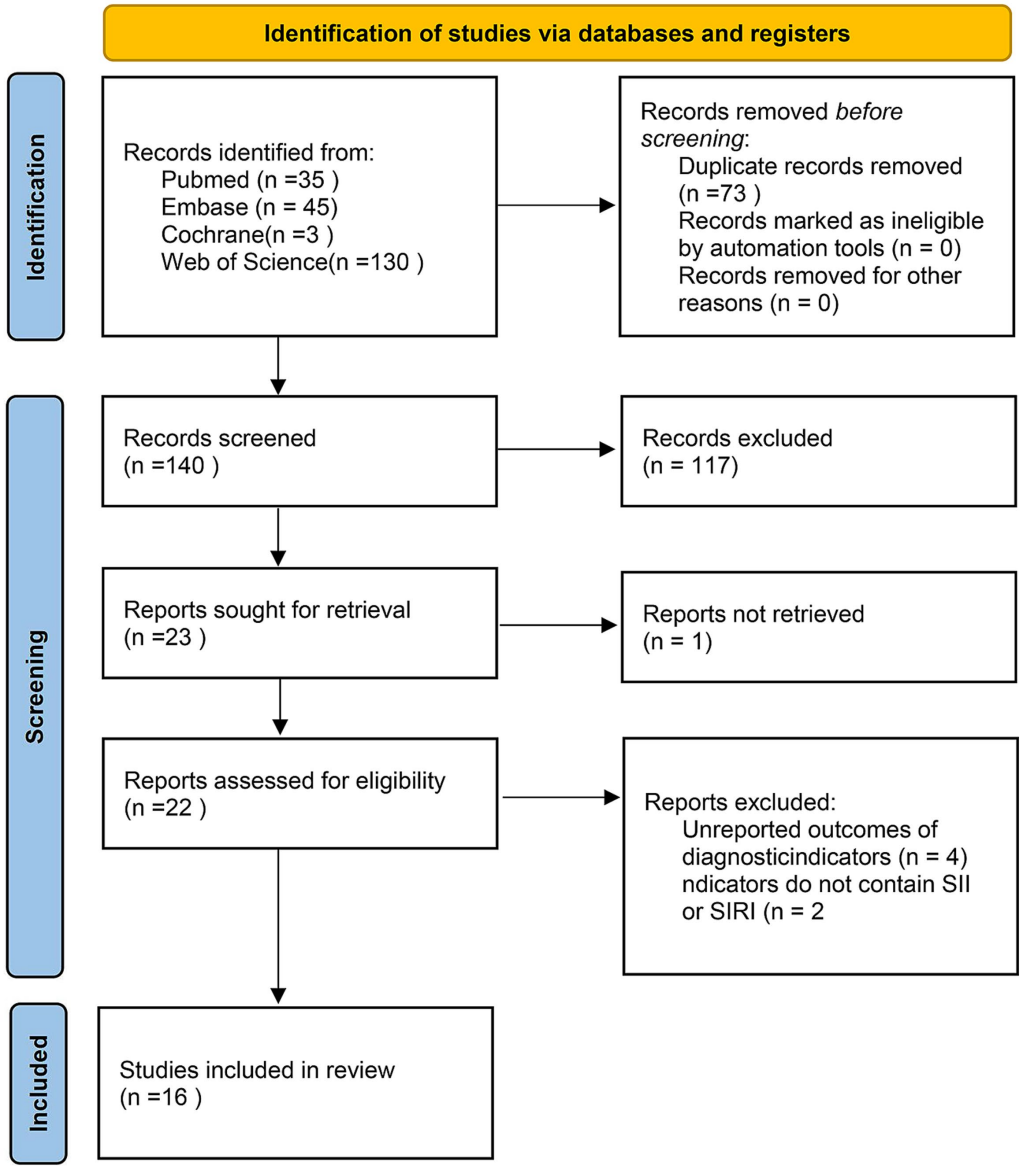
PRISMA flow diagram.

A total of 213 articles were obtained from the foregoing databases. Following topic screening by two independent researchers, 73 duplicate studies, 21 conference abstracts, eight reviews, four meta-analyses, six guidelines and letters, three animal studies, two case reports, and one article without full-text access were excluded. Additionally, 73 studies did not meet the inclusion criteria on populations. After the full texts were checked, six ineligible articles were further ostracized. Ultimately, 16 studies (25–40) were encompassed (Figure 1).

### 3.2 Eligible studies, characteristics and quality assessment

This study systematically included 16 retrospective studies (25–40) published between 2001 and 2024, involving a total of 4,510 adult patients with aSAH. The study populations were drawn from five countries: China (*n* = 8), South Korea (*n* = 3), Japan (*n* = 3), the United States (*n* = 2), and Australia (*n* = 1). The pooled mean age of patients was 56.52 ± 12.27 years, with 37.9% being male. Among these studies, nine (26, 27, 29–31, 35–37, 40) reported data on SII, while 11 (25, 26, 28, 29, 32–34, 37–40) included data on SIRI. Ten studies (26, 28, 29, 32–35, 37–39) reported 90-day poor outcomes, and six (30, 31, 33, 35, 38, 40) reported DCI. Fifteen studies (25, 26, 28–40) involved multivariable analyses to adjust for potential confounders. All studies employed either endovascular intervention or craniotomy. According to the NOS, all studies scored seven or eight, indicating that the encompassed studies were of high quality (Table 1).

**Table 1:** Baseline characteristics and quality assessment of eligible studies.

Author	Year of publication	Country	Patient source	Uni−/multi-variate	Age (mean ± SD)	Outcomes	Sample size (male)	NOS score
Yuhei Yoshimoto (25)	2001	Japan	Single center	Multivariate	56.0 ± 12.0	SIRI	103 (37)	8
Rajat Dhar (26)	2008	United States	Single center	Multivariate	55.9 ± 13.5	SIRI	246 (88)	7
Verena Rass (27)	2018	Austria	Single center	Multivariate (adjusted for age, sex, pneumonia, loss of consciousness, Hunt, and Hess grade.)	57.0 ± 14.8	SIRI	297 (115)	8
Masaaki Hokari (28)	2020	Japan	Single center	Multivariate	65.0 ± 14.9	SIRI	87 (29)	7
Peng Zhang (29)	2020	China	Single center	Multivariate	57.6 ± 10.2	SIRI	178 (62)	8
Liuwei Chen (30)	2021	China	Single center	Multivariate	59.3 ± 11.4	SII	333 (128)	7
Joseph R. Geraghty (31)	2021	United States	Single center	Multivariate (age, aneurysm location, diabetes mellitus, hyperlipidemia, as well as modified Fisher scale)	55.0 ± 12.6	SII	246 (88)	8
Fushu Luo (32)	2021	China	Single center	Multivariate	57.3 ± 11.8	SII	76 (31)	8
Seonyong Yun (33)	2021	Korea	Single center	Multivariate	56.4 ± 13.1	SIRI/SII	680 (216)	8
Zhaobo Nie (34)	2023	China	Single center	Multivariate (age, hypertension, WFNS grade 4–5, mFS grade 3–4, a Graeb score of 5–12, acute hydrocephalus, as well as treatment modality)	54.7 ± 11.1	SIRI/SII	543 (236)	7
Ho Jun Yi (35)	2023	Korea	Single center	Multivariate	58.7 ± 16.0	SIRI/SII	279 (79)	8
Yeonhu Lee (36)	2023	Korea	Single center	Multivariate	55.1 ± 12.6	SII	170 (65)	8
Yuyang Hou (37)	2023	China	Single center	Multivariate [model 2 plus variables linked to disease severity (GCS, Hunt-Hess, WFNS, and mFisher grades, ICH and IVH)]	55.6 ± 9.8	SIRI	350 (135)	8
Tu Li (38)	2024	China	Single center	Multivariate (multivariable adjusted: adjusted for age, female, hypertension, diabetes mellitus, currently smoking, posterior circulation, acute hydrocephalus, WFNS grade of 4–5, SEBES score of 3–4, mFS grade of 3–7, and treatment modalities.)	55.3 ± 10.5	SIRI	650 (279)	8
Qiong Zhao (39)	2024	China	Single center	Univariate	56.5 ± 6.4	SII	102 (68)	7
Xian Wang (40)	2024	China	Single center	Multivariate	59.0 ± 9.0	SIRI/SII	140 (53)	8

SIRI, Systemic Inflammation Response Index; SII, Systemic Immune-Inflammation Index; NOS, Newcastle-Ottawa Scale; SD, Standard Deviation.

### 3.3 Meta-analysis

#### 3.3.1 Analysis of SII

**Figure 2:**
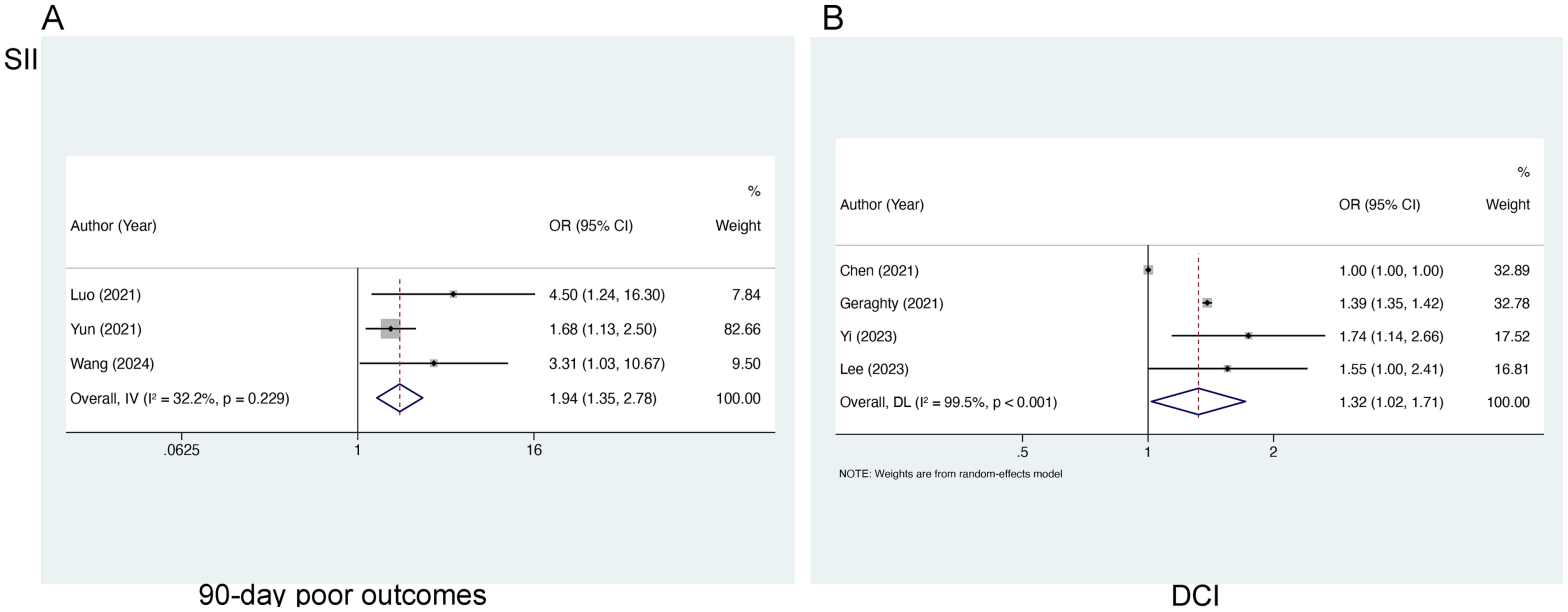
Meta-analysis of the association between SII and **(A)** 90-day poor outcomes and **(B)** DCI in patients with aSAH.

Three studies, comprising 896 patients, were encompassed in our analysis of SII in relation to 90-day poor outcomes. Statistical evaluation revealed no marked heterogeneity (I^2^ = 32.2%, *p* = 0.229), so a fixed-effects model was utilized. There existed a notable relation of elevated SII levels to 90-day AOs [OR = 1.94, 95% CI (1.35–2.78), *p* < 0.001] (Figure 2A), demonstrating that risen SII levels are linked to worse 90-day prognoses in the aSAH population. For the analysis of SII in relation to DCI, four studies concerning 1,028 patients were encompassed. A significant degree of heterogeneity was noted (I^2^ = 99.5%, *p* < 0.001), so a random-effects model was applied. The analysis demonstrated a marked link of higher SII levels to an elevated likelihood of DCI occurrence [OR = 1.32, 95% CI (1.02–1.71), *p* < 0.001] (Figure 2B).

#### 3.3.2 Analysis of SIRI

**Figure 3:**
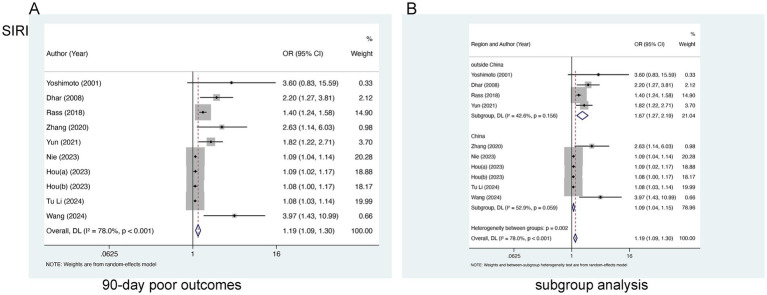
Meta-analysis of the association between SIRI and **(A)** 90-day poor outcomes and **(B)** subgroup analysis by country in patients with aSAH.

Nine studies encompassing 3,217 patients were incorporated into the evaluation of SIRI and 90-day poor outcomes. Marked heterogeneity was identified (I^2^ = 78.0%, *p* < 0.0001) (Figure 3A) and a random-effects model was employed. A notable link of elevated SIRI levels to 90-day poor outcomes was found [OR = 1.19, 95% CI (1.09–1.30), *p* < 0.001], suggesting the link of elevated SIRI levels to worse prognoses in aSAH patients. Subgroup analysis identified that the inclusion of study populations from China may contribute to the observed heterogeneity (I^2^ = 52.9 and 42.6%, respectively) (Figure 3B).

**Figure 4:**
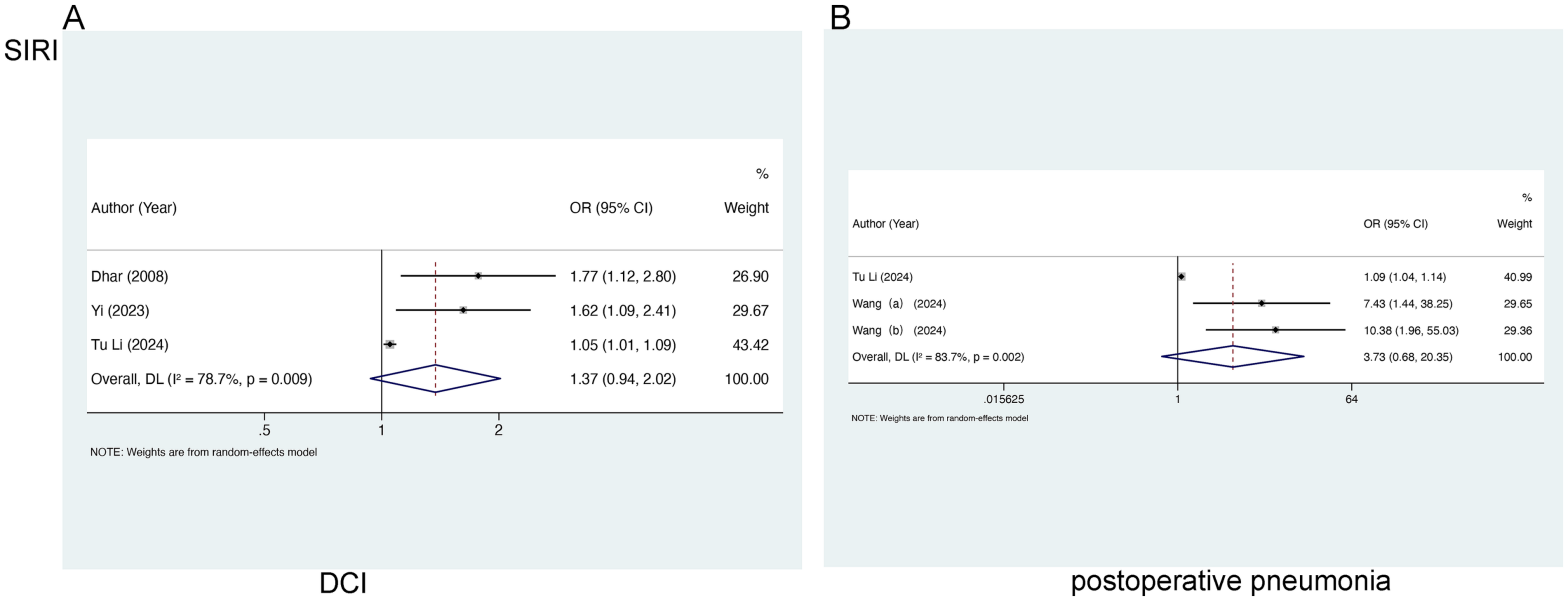
Meta-analysis of the association between SIRI and **(A)** DCI and **(B)** postoperative pneumonia in patients with aSAH.

For the analysis of SIRI in relation to DCI, three studies involving 1,205 patients were selected. Marked heterogeneity was noted (I^2^ = 78.7%, *p* = 0.009), so a random-effects model was leveraged. No marked relation of SIRI to DCI [OR = 1.37, 95% CI (0.94–2.02), *p* = 0.105] was found (Figure 4A). In the analysis of SIRI and postoperative pneumonia (POP), two studies involving 790 patients were included. Evident heterogeneity was noted (I^2^ = 83.7%, *p* = 0.002), and a random-effects model was adopted. A notable link of SII to POP [OR = 3.73, 95% CI (0.68–20.35), *p* = 0.128] was not observed (Figure 4B).

### 3.4 Sensitivity analysis

**Figure 5:**
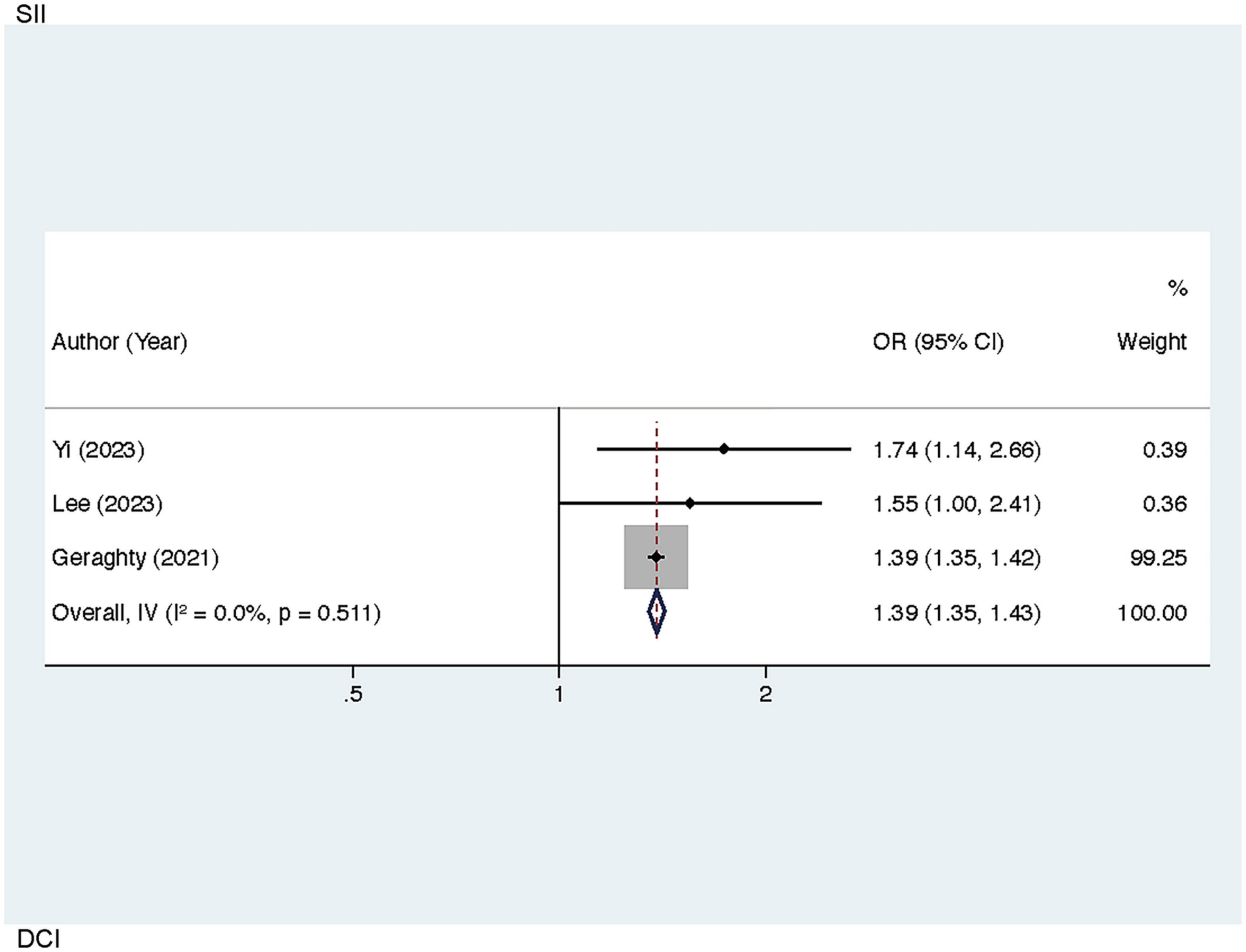
Sensitivity analysis of the association between SII and DCI in patients with aSAH (after excluding the study by Chen, 2021).

Sensitivity analyses helped to examine the robustness of the links of SII to worse 90-day outcomes, SII and DCI, and SIRI and worse 90-day outcomes. By sequentially excluding each individual study, the pooled results showed minimal variation from the original findings, indicating the stability of these associations. Notably, in the analysis of the correlation between SII and DCI, the exclusion of Chen (2021) led to a reduction in heterogeneity (I^2^ = 0.00%, *p* = 0.511), identifying this study as a source of heterogeneity. The recalculated pooled result yielded an OR of 1.39 [95% CI (1.35–1.43), *p* < 0.001] (Figure 5).

### 3.5 Publication bias

**Figure 6:**
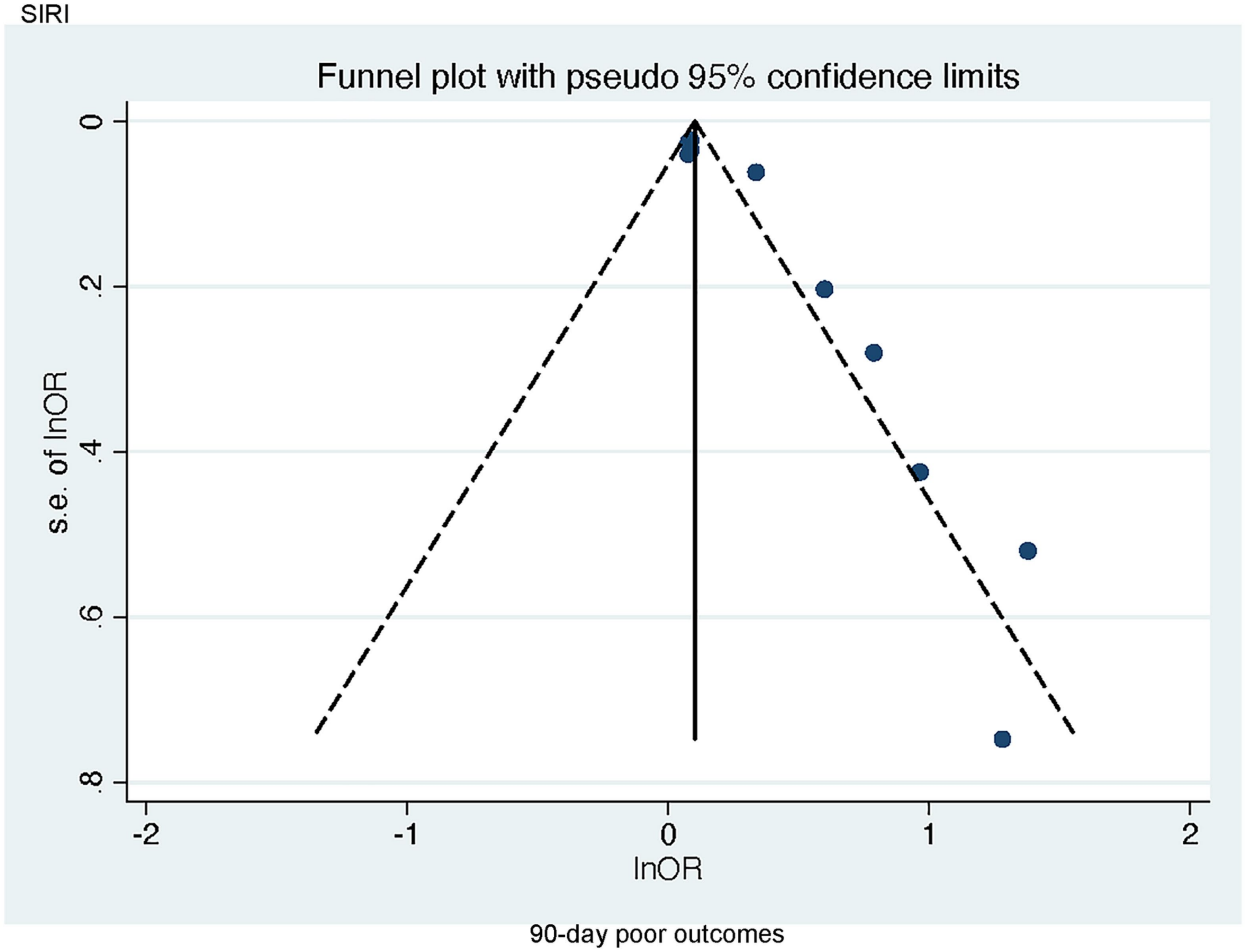
Funnel plot of the association between SIRI and 90-day poor outcomes in patients with aSAH.

Publication bias concerning the link of SIRI to poor 90-day outcomes was visually assessed through a funnel plot and further evaluated via Egger’s test, which demonstrated notable publication bias (*p* = 0.001 < 0.005) (Figure 6). Since there were limited studies examining the correlations of SII with poor 90-day outcomes as well as SII and DCI (*n* < 5), bias assessments were not performed for these outcomes.

### 3.6 Predictive value

#### 3.6.1 Predictive value of SII for 90-day AOs

**Figure 7:**
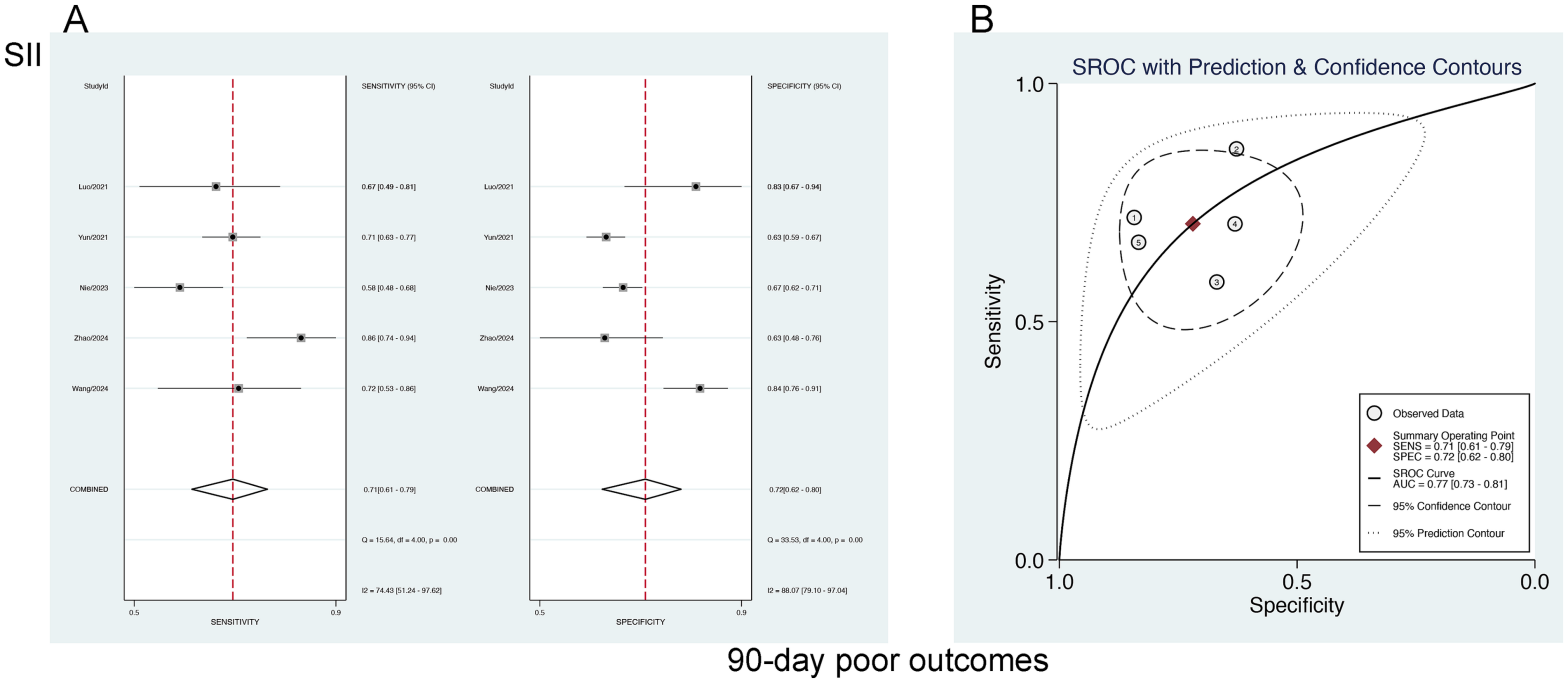
**(A)** Sensitivity and specificity of SII for predicting 90-day poor outcomes in patients with aSAH, and **(B)** the SROC curve.

A bivariate mixed-effects model was leveraged to ascertain the predictive performance of SII for poor 90-day outcomes. The results were a sensitivity of 0.71 [95% CI (0.61–0.79)], specificity of 0.72 [95% CI (0.62–0.80)] PLR of 2.51 [95% CI (1.80–3.50)], NLR of 0.41 [95% CI (0.30–0.56)], DOR of 3 [95% CI (2–4)], and an SROC-AUC of 0.77 [95% CI (0.73–0.81)]. SIRI demonstrated a certain predictive value for poor 90-day outcomes. Marked publication bias was not noted (*p* = 0.08) (Figure 7; Supplementary Figure S1). The evaluation of the diagnostic cutoff revealed no notable threshold effect (Spearman correlation coefficient = 0.300, *p* = 0.624). Subgroup analysis according to the share of male participants (>50% vs. <50%) showed higher specificity in studies with >50% male participants (*p* = 0.94) (Supplementary Table S2).

#### 3.6.2 Predictive value of SII for DCI

**Figure 8:**
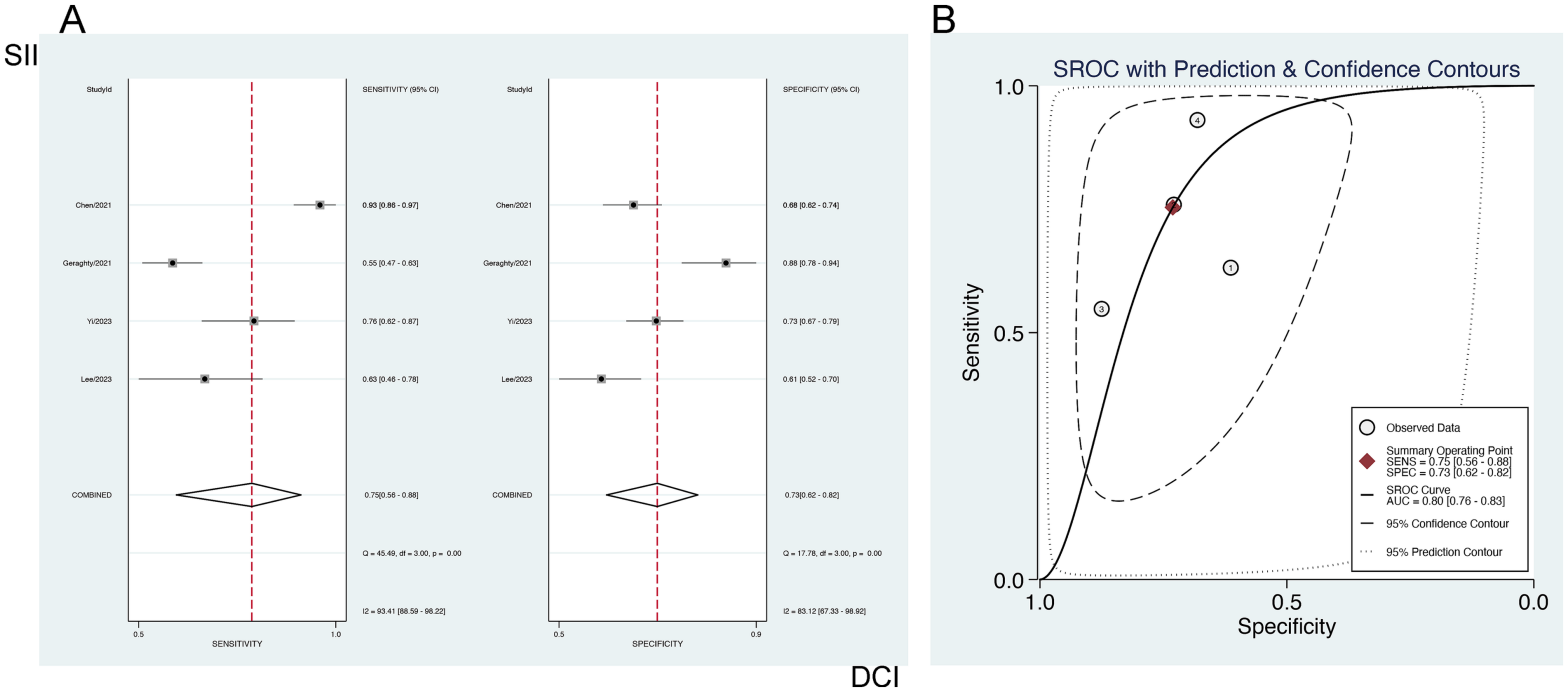
Sensitivity and specificity of SII for predicting DCI in patients with aSAH **(A)**, and **(B)** the SROC curve.

The forecasting significance of SII for DCI was examined via a bivariate mixed-effects model. Our analysis showed a sensitivity of 0.75 [95% CI (0.56–0.88)], specificity of 0.73 [95% CI (0.62–0.82)], PLR of 2.80 [95% CI (2.02–3.89)], NLR of 0.34 [95% CI (0.18–0.63)], DOR of 3 [95% CI (2–4)], and an SROC-AUC of 0.80 [95% CI (0.76–0.83)], demonstrating the predictive value of SII for DCI. Significant publication bias was observed (*p* = 0.05) (Figure 8; Supplementary Figure S2). The analysis of diagnostic thresholds displayed no marked threshold effect (Spearman correlation coefficient = 0.400, *p* = 0.600). Subgroup analysis based on the study region (South Korea vs. others) showed no marked differences (*p* > 0.01) (Supplementary Table S2).

#### 3.6.3 Predictive value of SIRI for 90-day AOs

**Figure 9:**
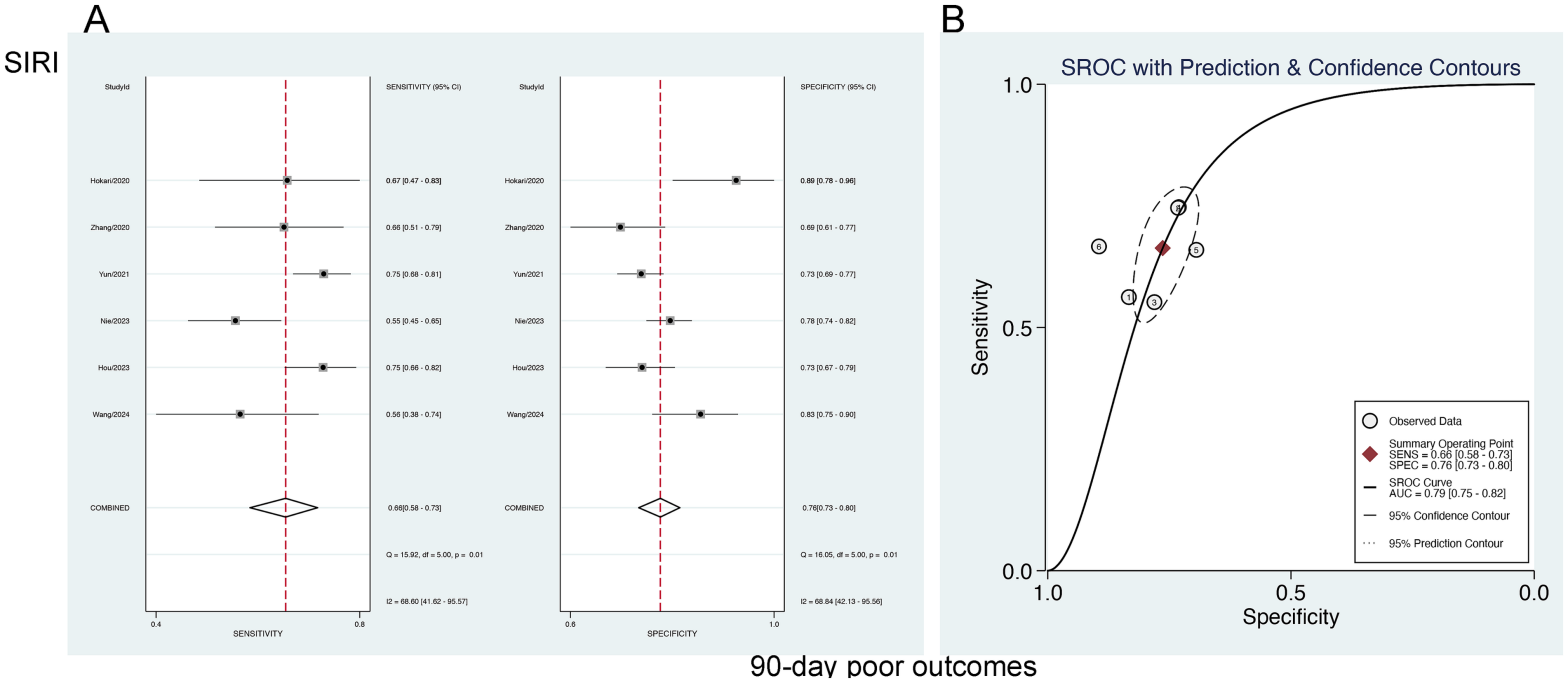
Sensitivity and specificity of SIRI in predicting 90-day poor outcomes in patients with aSAH **(A)**, and the SROC curve **(B)**.

The forecasting significance of SIRI for poor 90-day outcomes was analyzed utilizing a bivariate mixed-effects model. This analysis revealed a sensitivity of 0.66 [95% CI (0.58–0.73)], specificity of 0.76 [95% CI (0.73–0.80)], PLR of 2.81 [95% CI (2.49–3.17)], NLR of 0.44 [95% CI (0.36–0.54)], DOR of 3 [95% CI (2–4)], as well as an SROC-AUC of 0.79 [95% CI (0.75–0.82)], showing the predictive value for 90-day AOs. Potential publication bias was suggested (*p* = 0.89) (Figure 9; Supplementary Figure S3). No evident threshold effect (Spearman correlation coefficient = 0.314, *p* = 0.544) was observed. Subgroup analysis based on study region (China vs. non-China) revealed higher specificity in non-China studies compared to those conducted in China (*p* < 0.001) (Supplementary Table S2).

## 4 Discussion

aSAH makes up approximately 5% of all stroke cases and represents a devastating condition that greatly endangers human life and health (41). It features a high mortality rate and an unfavorable prognosis (42). The development of effective predictive tools can enable clinicians to swiftly tailor and implement optimal therapeutic strategies for each patient. Therefore, identifying key prognostic factors influencing aSAH outcomes has been a focal point of clinical research. In recent years, clinical research has increasingly focused on the inflammatory indices SIRI and SII, achieving notable progress. Both SII and SIRI, calculated from routine blood count data, offer significant clinical utility: by using blood tests obtained at admission and during hospitalization in patients with aSAH, readily available and rapidly reported results can be transformed into prognostic assessment tools early in the disease course without requiring additional testing. Although many studies have explored the association of SIRI and SII with aSAH outcomes, the results have been inconsistent, and perspectives on their clinical application remain divergent. Therefore, larger-scale studies are still needed to further validate their clinical value. Our meta-analysis incorporated 16 studies, encompassing 4,510 individuals. The SII was demonstrated as an independent prognostic factor for 90-day AOs and DCI in the aSAH population. Additionally, the SIRI can independently forecast worse 90-day prognosis in aSAH sufferers, with both indices exhibiting moderate predictive efficacy.

The intracranial aneurysm formation and rupture involve plenty of factors, commonly attributed to hypertension, lipid accumulation, atherosclerosis, smoking-related vascular injury, hemodynamic stress at arterial bifurcations, and a combination of genetic and susceptibility factors (43). The pathological mechanisms leading to brain injury in aSAH are activated at the moment of aneurysmal rupture, encompassing acute mechanical damage and hemorrhage-triggered vascular injury. Early brain injury, typically manifesting within the initial 72-h period after the onset of hemorrhage, is closely linked to DCI development and progression, and long-term prognosis and death rates (8, 44, 45).

SII and SIRI were established to enhance predictive capabilities by integrating various peripheral blood cell markers, including platelets, neutrophils, lymphocytes, and monocytes (46). The pathophysiological process of aSAH is highly complex, often inducing a systemic inflammatory response coupled with immunosuppression (47, 48). Clinical data have indicated that elevated leukocyte counts in peripheral blood are positively correlated with stroke severity and poor clinical outcomes (6, 49, 50). At ischemic locations, neutrophils release inflammatory factors like matrix metalloproteinase-9 following aSAH-related DCI, with higher concentrations and counts exacerbating brain tissue and blood–brain barrier damage. Impairment of the blood–brain barrier enhances the infiltration of leukocytes, resulting in severe consequences like brain swelling, hemorrhage, and neurological decline (46). Studies have shown that specific lymphocyte subsets such as CD4 + and CD8 + T cells, release cytotoxic and pro-inflammatory factors, including interleukin-17 (IL-17) and interferon-gamma (IFN-*γ*), which trigger inflammatory responses and give rise to brain injury (51). Upon infiltration into ischemic regions, peripheral monocytes differentiate into macrophages with either pro-inflammatory or anti-inflammatory phenotypes. Pro-inflammatory macrophages exacerbate inflammatory processes and tissue damage, whereas protective macrophages mitigate ischemic damage, boost angiogenesis, and enable inflammation resolution (52). Moreover, a significant reduction in peripheral blood T lymphocytes has been noted at the acute SAH stage (53), with lymphopenia potentially linked to the risen risk of pneumonia and higher mortality rates within 3 months post-onset (53, 54). Elevated platelets during aSAH is also related to DCI and poor outcomes, whereas antiplatelet therapy can lower the occurrence of DCI (55, 56). Therefore, neutrophils, platelets, and lymphocytes are closely linked to the prevalence of complications and prognosis in the aSAH population.

Previous studies have underscored the close association between early systemic inflammatory response following aSAH and adverse patient outcomes. The alterations in early systemic inflammation involve many inflammatory biomarkers like leukocytes, the neutrophil-to-lymphocyte ratio (NLR), the monocyte-to-lymphocyte ratio (MLR), and the SII (57, 58). Certain investigators have employed ratio-based indicators, including NLR and MLR, to forecast outcomes of aSAH individuals (59–61). Our meta-analysis indicates a clear connection of SII with poor prognosis at 90 days; however, due to the limited data derived from only three studies, our findings should be approached with careful consideration, as additional studies are necessary to enhance their robustness.

DCI following aSAH is characterized by delayed neurological deterioration resulting from infarction and the progression of cerebral vasospasm (62). Although the exact mechanisms are not clear, factors like vasoconstriction, microspasm, microthrombosis, procoagulant activity, as well as endothelial dysfunction are recognized as contributors to DCI progression (63, 64). Significant heterogeneity was detected in our pooled analysis of the relationship between SII and DCI. Further investigation identified substantial discrepancies between the findings of Chen (2021) and those of other studies, which may be attributable to differences in study regions. Specifically, Chen (2021) focused on a Chinese cohort, whereas Yi (2023), Lee (2023), and Geraghty (2021) examined non-Chinese populations. Future studies incorporating more diverse regions and larger sample sizes are necessitated to explore this relationship further.

The SIRI, which integrates neutrophils, monocytes, and lymphocytes, serves as a comprehensive prognostic marker reflecting the host’s immune and inflammatory status (65–67). Neutrophils can amplify inflammation through various inflammatory mediators and mechanisms (68, 69). Peripheral blood monocytes also reflect underlying inflammatory conditions (70). Moreover, lymphocytes, as critical components of immune surveillance and inflammation resolution, are inversely associated with persistent inflammation (71). Therefore, elevated SIRI reflects a pro-inflammatory hyperactivity mediated by increased neutrophils and monocytes, coupled with suppressed anti-inflammatory responses due to lymphocyte reduction (58). This imbalance ultimately causes secondary brain injury and unfavorable prognosis in aSAH individuals. Our meta-analysis identifies SIRI as an independent predictor of unfavorable 90-day outcomes. Furthermore, subgroup analysis based on geographic origin suggests that study populations from China may represent a potential source of heterogeneity (Figure 5; Supplementary Table S2). This finding implies that the correlation and predictive efficacy of SIRI might be more pronounced in non-Chinese populations. Nevertheless, more studies are necessary to verify the foregoing through expanded analyses.

Accurate prognostic biomarkers greatly help clinicians detect high-risk patients, thereby allowing for the prioritization and provision of dedicated care for better outcomes. Therefore, our meta-analysis bears clinical significance, as this is the first study unraveling and analyzing the prognostic relevance of SIRI and SII in aSAH individuals. Our study presents fresh insights into the forecasting of patient prognosis, the evaluation of prognostic value, and implications for future studies. However, some limitations should be acknowledged. First, although the original studies all provided sROC curves, they did not report the specific values corresponding to different thresholds, and the limited number of included studies precluded conducting threshold-related meta-regression analyses. Second, the assessment criteria, cut-off values, and prognostic scales for SII and SIRI were not standardized, which may have affected the pooled results. Third, related outcomes such as POP and deep vein thrombosis (DVT) were reported in only a few studies, limiting further analysis. Fourth, this study included only English-language publications, which may introduce selection bias. Fifth, the included studies generally did not provide detailed information on aneurysm location or family history, preventing related statistical analyses. Finally, the majority of the included studies were conducted in Asia, and data from other regions (e.g., Europe, Africa, South America) remain scarce. Future research should report more detailed threshold-related data, include key prognostic factors, and expand to diverse regions and populations to enhance the reliability and generalizability of the findings.

## 5 Conclusion

Our meta-analysis reveals SII and SIRI as independent predictors of 90-day AOs in the aSAH population, with the former also proved as an independent predictor of DCI in them. Both indices exhibit a certain degree of predictive accuracy. Large-scale, prospective studies across multiple regions and populations are necessary in the future, with improved collection and reporting of clinical data, to further corroborate the reliability of this meta-analysis.

## Data Availability

The original contributions presented in the study are included in the article/Supplementary material, further inquiries can be directed to the corresponding author.
