# Supplementary material for: Association between systemic immune-inflammation index, systemic inflammation response index, and adverse outcomes in aneurysmal subarachnoid hemorrhage: a meta-analysis
Source: Front Neurol. 2025 Oct 6;16:1596126. doi: 10.3389/fneur.2025.1596126 (PMC12535894; doi:10.3389/fneur.2025.1596126)
Supplement: Supplementary file 1 [file Table_1.docx]

**Table S1 Retrieval strategy**

| **Pubmed: 35 records** | #1: (Subarachnoid Hemorrhage[Title/Abstract] OR Subarachnoid Hemorrhage*[Title/Abstract]) OR SAH[Title/Abstract] OR SAHs[Title/Abstract] OR Aneurysmal Subarachnoid Hemorrhage[Title/Abstract] OR Aneurysmal Subarachnoid Hemorrhages[Title/Abstract] OR aneurysmal subarachnoid haemorrhage[Title/Abstract] OR aSAH[Title/Abstract] OR subarachnoid bleeding[Title/Abstract] OR subarachnoid haemorrhage[Title/Abstract] OR subarachnoid hemorrhagia[Title/Abstract] OR subarachnoidal bleeding[Title/Abstract] OR  subarachnoidal haemorrhage[Title/Abstract] OR subarachnoidal hemorrhage[Title/Abstract] OR ruptured intracranial aneurysm[Title/Abstract] OR ruptured cerebral aneurysm[Title/Abstract] OR  ruptured brain aneurysm[Title/Abstract] OR subarachnoid blood[Title/Abstract])  #2: (systemic immune inflammation index[Title/Abstract] OR systemic inflammation response index[Title/Abstract] OR systemic immune-inflammation index[Title/Abstract] OR systemic immune-inflammatory index[Title/Abstract] OR Systemic Immunity-inflammation Index[Title/Abstract] OR systemic inflammation reaction index[Title/Abstract] OR systemic inflammatory reaction index[Title/Abstract] OR Systemic Inflammatory Response Index[Title/Abstract] OR SII[Title/Abstract] OR SIRI[Title/Abstract] OR  systemic immune inflammatory index[Title/Abstract] OR systemic-immune-inflammation index[Title/Abstract] )  #3: #1 AND #2 |
| --- | --- |
| **Embase: 45 records** | #1：'Subarachnoid Hemorrhage':ab,ti OR 'Subarachnoid Hemorrhage*[Title/Abstract]) OR SAH':ab,ti OR 'SAHs':ab,ti OR 'Aneurysmal Subarachnoid Hemorrhage':ab,ti OR 'Aneurysmal Subarachnoid Hemorrhages':ab,ti OR 'aneurysmal subarachnoid haemorrhage':ab,ti OR 'aSAH':ab,ti OR 'subarachnoid bleeding':ab,ti OR 'subarachnoid haemorrhage':ab,ti OR 'subarachnoid hemorrhagia':ab,ti OR 'subarachnoidal bleeding':ab,ti OR '  subarachnoidal haemorrhage':ab,ti OR 'subarachnoidal hemorrhage':ab,ti OR 'ruptured intracranial aneurysm':ab,ti OR 'ruptured cerebral aneurysm':ab,ti OR '  ruptured brain aneurysm':ab,ti OR 'subarachnoid blood ':ab,ti  #2：'systemic immune inflammation index':ab,ti OR 'systemic inflammation response index':ab,ti OR 'systemic immune-inflammation index':ab,ti OR 'systemic immune-inflammatory index':ab,ti OR 'Systemic Immunity-inflammation Index':ab,ti OR 'systemic inflammation reaction index':ab,ti OR 'systemic inflammatory reaction index':ab,ti OR 'Systemic Inflammatory Response Index':ab,ti OR 'SII':ab,ti OR 'SIRI':ab,ti OR '  systemic immune inflammatory index':ab,ti OR 'systemic-immune-inflammation index ':ab,ti  #3: #1 AND #2 |
| **Cochrane Library: 3 records** | #1：'Subarachnoid Hemorrhage':ab,ti OR 'Subarachnoid Hemorrhage*[Title/Abstract]) OR SAH':ab,ti OR 'SAHs':ab,ti OR 'Aneurysmal Subarachnoid Hemorrhage':ab,ti OR 'Aneurysmal Subarachnoid Hemorrhages':ab,ti OR 'aneurysmal subarachnoid haemorrhage':ab,ti OR 'aSAH':ab,ti OR 'subarachnoid bleeding':ab,ti OR 'subarachnoid haemorrhage':ab,ti OR 'subarachnoid hemorrhagia':ab,ti OR 'subarachnoidal bleeding':ab,ti OR '  subarachnoidal haemorrhage':ab,ti OR 'subarachnoidal hemorrhage':ab,ti OR 'ruptured intracranial aneurysm':ab,ti OR 'ruptured cerebral aneurysm':ab,ti OR '  ruptured brain aneurysm':ab,ti OR 'subarachnoid blood ':ab,ti  #2：'systemic immune inflammation index':ab,ti OR 'systemic inflammation response index':ab,ti OR 'systemic immune-inflammation index':ab,ti OR 'systemic immune-inflammatory index':ab,ti OR 'Systemic Immunity-inflammation Index':ab,ti OR 'systemic inflammation reaction index':ab,ti OR 'systemic inflammatory reaction index':ab,ti OR 'Systemic Inflammatory Response Index':ab,ti OR 'SII':ab,ti OR 'SIRI':ab,ti OR '  systemic immune inflammatory index':ab,ti OR 'systemic-immune-inflammation index ':ab,ti  #3: #1 AND #2 |
| **WOS: 130 records** | #1: TS=(Subarachnoid Hemorrhage OR Subarachnoid Hemorrhage* OR SAH OR SAHs OR Aneurysmal Subarachnoid Hemorrhage OR Aneurysmal Subarachnoid Hemorrhages OR aneurysmal subarachnoid haemorrhage OR aSAH OR subarachnoid bleeding OR subarachnoid haemorrhage OR subarachnoid hemorrhagia OR subarachnoidal bleeding OR subarachnoidal haemorrhage OR subarachnoidal hemorrhage OR spontaneous hemorrhage OR ruptured intracranial aneurysm OR ruptured cerebral aneurysm OR ruptured brain aneurysm OR subarachnoid blood)  #2: TS=( systemic immune inflammation index OR systemic inflammation response index OR systemic immune-inflammation index OR systemic immune-inflammatory index OR Systemic Immunity-inflammation Index OR systemic inflammation reaction index OR systemic inflammatory reaction index OR Systemic Inflammatory Response Index OR SII OR SIRI OR systemic immune inflammatory index OR systemic-immune-inflammation index)  #3: #1 AND #2 |
