# Supplementary material for: Association between systemic immune-inflammation index, systemic inflammation response index, and adverse outcomes in aneurysmal subarachnoid hemorrhage: a meta-analysis
Source: Front Neurol. 2025 Oct 6;16:1596126. doi: 10.3389/fneur.2025.1596126 (PMC12535894; doi:10.3389/fneur.2025.1596126)
Supplement: Supplementary file 2 [file Table_2.docx]

Table S2 Subgroup analysis of SII and SIRI in predicting poor 90-day prognosis and SII in predicting DCI in patients with aSAH

| Outcomes | Parameter | Category | Number of Studies | Sensitivity | p1 | Specificity | p2 |
| --- | --- | --- | --- | --- | --- | --- | --- |
| SII for predicting a poor 90-day prognosis in aSAH patients | Over 50% of males | Yes | 4 | 0.67[0.60-0.74] | <0.001 | 0.74[0.64-0.83] | 0.94 |
|  |  | No | 1 | 0.86[0.76-0.96] |  | 0.63[0.40-0.86] |  |
| SII for predicting DCI in aSAH patients | Korea | Yes | 2 | 0.71[0.45-0.96] | 0.53 | 0.68[0.55-0.81] | 0.04 |
|  |  | No | 2 | 0.79[0.59-0.99] |  | 0.79[0.68-0.90] |  |
| SIRI for predicting a poor 90-day prognosis in aSAH patients | China | Yes | 4 | 0.64[0.55-0.73] | 0.05 | 0.76[0.71-0.81] | <0.001 |
|  |  | No | 2 | 0.70[0.59-0.81] |  | 0.78[0.70-0.86] |  |
